# Effects of feeding *Saccharomyces cerevisiae* fermentation products on the health and growth performance of Holstein dairy calves

**DOI:** 10.3168/jdsc.2021-0172

**Published:** 2022-03-16

**Authors:** R.N. Klopp, R.E. Centeno-Martinez, I. Yoon, T.A. Johnson, J.P. Boerman

**Affiliations:** 1 Department of Animal Sciences, Purdue University, West Lafayette, IN 47907; 2 Diamond V, Cedar Rapids, IA 52404

## Abstract

It is essential to reduce antibiotic use in the livestock industry, which leads to a need for alternatives to antibiotics that reduce illness and promote growth in dairy calves. The objective of this study was to evaluate the effect of feeding dairy calves *Saccharomyces cerevisiae* fermentation products (SCFP) on average daily gain (ADG) and antibiotic use in dairy calves through 4 mo of age. Holstein bull calves (n = 60; 5 ± 3 d old) were blocked by body weight (BW) and serum total protein (STP) and assigned to 1 of 2 treatments. The control treatment (CON) fed a 24% crude protein (CP):17% fat milk replacer (MR), calf starter, grower #1, and grower #2 with no SCFP added. The SCFP treatment fed the same MR with 1 g/d of SCFP, calf starter with 0.8% (dry matter; DM) SCFP, grower #1 with 0.44% (DM) SCFP, and grower #2 with 0.275% (DM) SCFP. Calves were offered 2.84 L (12.5% solids) of MR twice daily (0630 and 1630 h) through d 51 and MR once daily (0630 h) from d 52 to 56, and were weaned on d 57. From d 1 to 56, calves also received ad libitum access to calf starter and water. On d 57, calves were switched to grower #1 and on d 84, calves were switched to grower #2, which contained a lower level of CP and a higher level of neutral detergent fiber (NDF). Individual calf BW, body condition score (BCS), hip height (HH), and hip width (HW) were measured biweekly from d 0 to 112. Feed intake was recorded daily, and feed efficiency (gain:feed) and ADG were calculated. Daily fecal and respiratory scores were recorded for each calf through d 56, and all medical interventions were recorded for the duration of the study and grouped based on illness. We found no effect of treatment on STP, BW, BCS, HH, or HW at d 0 or 56, nor effects on preweaning ADG and feed efficiency. No treatment effect was observed for BCS or HH at d 112; however, BW and HW were increased in SCFP calves at d 112. A treatment tendency was observed for postweaning ADG, with SCFP calves being larger than CON calves and SCFP calves having improved feed efficiency compared with CON calves after weaning. A treatment effect was observed for respiratory treatments postweaning, with SCFP calves being treated less frequently than CON calves. Our results suggest that feeding SCFP to calves improves postweaning growth and feed efficiency, and reduces postweaning respiratory disease interventions.

## Highlights

• Feeding dairy calves SCFP in milk replacer and solid feeds until 4 mo improved postweaning ADG and feed efficiency.
• SCFP reduced respiratory illness antibiotic treatments in calves.
• Feeding SCFP to calves did not affect daily fecal scores or preweaning growth.

## Graphical Abstract

**Figure:**
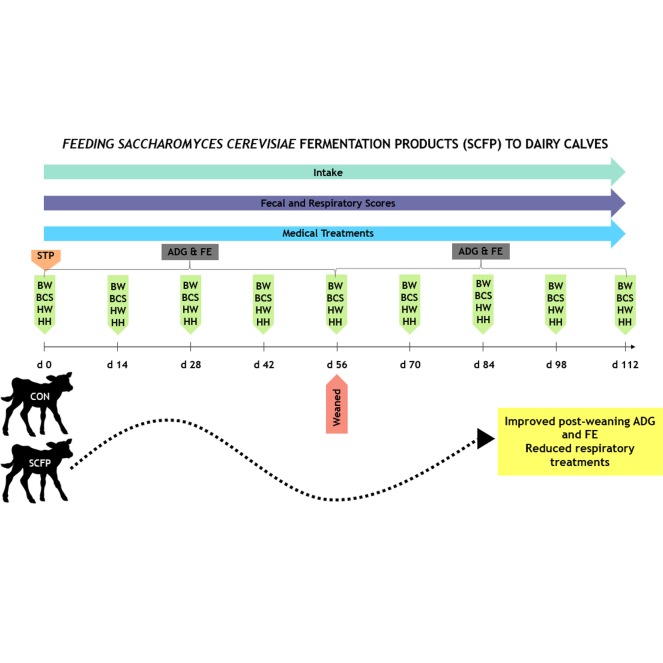
**Summary:** The goal of this study was to determine the effects of feeding *Saccharomyces cerevisiae* fermentation products (SCFP) on the health and performance of dairy calves. Calves either received SCFP in both their milk replacer and solid feeds or they did not receive SCFP (CON). To evaluate health and performance, serum total protein (STP) was measured at the start of the study; body weight (BW), body condition score (BCS), hip width (HW), and hip height (HH) were measured biweekly; average daily gain (ADG) and feed efficiency (FE) were calculated pre- and postweaning; and intake, medical treatments, respiratory scores, and fecal scores were recorded daily. We determined that feeding SCFP to calves improved both health and performance as observed by increased postweaning ADG and FE, as well as reduced respiratory treatments.

The goal of calf feeding systems is to provide calves with optimum nutrition to promote growth, health, and future milk production. According to nationwide survey studies conducted on US dairy farms, digestive problems affect 20 to 25% of preweaning calves, and pneumonia affects 5 to 18% (NAHMS, 2012; Walker et al., 2012). Of the preweaning calves affected by digestive illnesses, 72 to 83% were treated with antibiotics, and of those affected by pneumonia, 90 to 100% were treated with antibiotics (NAHMS, 2012; Walker et al., 2012). After weaning, respiratory disease affects 11% of heifers, where 82% of those affected receive antibiotics (NAHMS, 2012). Calf disease shifts energy from growth to the immune system, leading to reduced performance and increasing the risk of calf mortality, both of which have a negative economic impact on producers (Stanton et al., 2012; Windeyer et al., 2014). Additionally, reducing antibiotic treatments and incidence of illness and improving growth in calves before weaning positively affects first-lactation milk production (Heinrichs and Heinrichs, 2011; Stanton et al., 2012; Soberon and Van Amburgh, 2013; Gelsinger et al., 2016).

In the livestock industry, antibiotic use has come under recent scrutiny because of the increasing prevalence of antibiotic-resistant bacteria (Langford et al., 2003; Loo et al., 2019). This has led to the need for alternatives to antibiotics that can improve calf health and thus reduce reliance on antibiotics. *Saccharomyces cerevisiae* fermentation products (**SCFP**) is the term used to describe the products from the anaerobic fermentation of *Saccharomyces cerevisiae*, and includes compounds such as B vitamins, antioxidants, phytosterols, short-chain fatty acids, and organic acids, among others. Research evaluating the effects of SCFP on calf health and growth suggests that SCFP decreases the incidence of diarrhea (Brewer et al., 2014; Alugongo et al., 2017) and increases DMI and BW gain (Lesmeister et al., 2004). However, much of the research evaluating the effects of feeding SCFP to calves on growth and health has focused on the preweaning phase and scouring. The goal of this study was to evaluate the effects of SCFP on postweaning growth and respiratory disease in calves.

The primary objective of this study was to evaluate the effects of SCFP, when supplemented in MR, calf starter, and calf grower, on growth in dairy calves through 4 mo of age. The secondary objectives of this study included the effects of SCFP on intake, feed efficiency (**FE**), and respiratory disease until 4 mo of age in dairy calves. Our hypothesis was that supplementing calves with SCFP would lead to improved growth and FE and reduced incidences of respiratory disease and antibiotic treatment.

All procedures involving animals were reviewed and approved by the Institutional Animal Care and Use Committee at Purdue University (Protocol #1808001783). Sixty Holstein bull calves, 5 ± 3 d of age (mean ± SD), were received in 2 separate batches (n = 30 calves per batch; 15 calves per treatment per batch) on May 24 and September 13, 2019, from a dairy farm 55 km from the Purdue University Animal Sciences Research and Education Center (**ASREC**). Calves were transported by a trailer (2.3 × 7.3 m; Wilson) to ASREC, where they were placed in individual hutches (length × width × height; 212 cm × 114 cm × 122 cm; Calf-Tel) with a fenced-in outside area (3.5 × 1.2 m). Individual hutches were bedded with wood shavings and rebedded as needed. On d 59 of the study, calves were moved from individual hutches to group hutches (208 cm × 259 cm × 180 cm; Calf-Tel) with a fenced-in outside area (5.2 × 2.6 m). Group hutches were bedded with wood shavings and straw, as needed, depending on the temperature and moisture (6 total group hutches, 4–5 calves each).

Before arrival (d −1) at the farm of origin, a 10-mL blood sample was taken from each calf via the jugular vein, allowed to clot for 2 h, and then centrifuged at 3,100 × *g* for 20 min to evaluate serum total protein (**STP**) for passive transfer of immunity using a refractometer (LW Scientific). Calves were randomly assigned to 1 of 2 treatments (n = 60; 30 calves per treatment) upon arrival at ASREC. Calves on the control treatment (**CON**) were fed a 24% CP:17% fat milk replacer (**MR**), calf starter, grower #1, and grower #2 with no SCFP added. Calves on the SCFP treatment were fed 24% CP:17% fat MR with 1 g/d of SCFP, calf starter with 0.8% (DM) SCFP, grower #1 with 0.44% (DM) SCFP from d 57 to 84, and grower #2 with 0.275% (DM) SCFP from d 85 to 112. Calves were received in 2 separate batches, and each batch was blocked by BW. The average initial BW for batch 1 calves was 45.2 ± 4.7 kg, whereas the average initial BW was 44.8 ± 4.2 kg for batch 2 calves. Calves from each batch (n = 30) were blocked into low BW (block 1, n = 10), intermediate BW (block 2, n = 10), and high BW (block 3, n = 10) and then randomly assigned to treatment (CON vs. SCFP) within each block.

Calves were offered 2.84 L (12.5% solids) of MR twice daily (0630 and 1630 h) through d 51 of the study; then, from d 52 to 56, calves were fed MR once daily (0630 h) and weaned on d 57. Refusals were recorded daily and calves with more than a 0.95-L refusal of MR were fed the remainder of the milk using an esophageal tube feeder (Nasco). All calves received the same MR powder (Table 1). For the SCFP calves, 15 g of SmartCare (Diamond V) was added to 150 mL of MR and mixed thoroughly, and each SCFP bottle received 10 mL of the SCFP mixture solution.

**Table 1:** Chemical composition (% of DM unless otherwise noted) of milk replacer (MR), starter, and grower for control (CON) and *Saccharomyces cerevisiae* fermentation products (SCFP) experimental treatment diets^1^

Item	MR (n = 8)	CON Starter (n = 8)	SCFP Starter^2^ (n = 8)	CON Grower #1^3^ (n = 4)	SCFP Grower #1^3^, ^4^ (n = 4)	CON Grower #2^5^ (n = 4)	SCFP Grower #2^5^, ^6^ (n = 4)
DM, %	96.6 ± 0.1	86.6 ± 0.3	86.6 ± 0.5	88.8 ± 0.3	88.5 ± 0.4	89.0 ± 0.7	89.4 ± 0.7
CP	25.2 ± 0.3	23.7 ± 2.2	21.7 ± 1.2	20.5 ± 0.3	20.5 ± 0.3	18.3 ± 0.2	18.4 ± 0.1
Fat	17.8 ± 0.2	3.6 ± 0.2	3.4 ± 0.6	3.7 ± 0.1	3.9 ± 0.2	4.1 ± 1.1	3.8 ± 0.6
ADF	—	10.7 ± 0.7	10.3 ± 0.5	11.4 ± 0.8	10.9 ± 0.8	21.0 ± 1.7	22.6 ± 1.0
aNDF^7^	—	18.4 ± 0.5	19.0 ± 1.1	30.8 ± 1.2	29.5 ± 1.1	40.3 ± 2.8	42.3 ± 1.4
Ash	8.7 ± 0.1	7.8 ± 0.8	7.3 ± 0.5	7.9 ± 0.2	7.7 ± 0.2	5.7 ± 0.7	6.2 ± 0.7

1 CON = MR, calf starter, and calf growers with no SCFP added; SCFP = MR with 1 g/d of SmartCare (Diamond V), calf starter with NutriTek (Diamond V), and calf growers with NutriTek.

2 Contained 0.8% NutriTek (DM).

3 Fed from d 57 to 84.

4 Contained 0.44% NutriTek (DM).

5 Fed from d 85 to 112.

6 Contained 0.275% NutriTek (DM).

7 aNDF = ash-free neutral detergent fiber.

From d 1 to 56, calves received ad libitum access to a texturized calf starter and water. Individual starter intake was recorded daily. Starting on d 57, calves were switched to calf grower #1 (CON, no SCFP added; SCFP, 0.44% SCFP added), and on d 85, calves were switched to calf grower #2 (CON, no SCFP added; SCFP, 0.275% SCFP added). The reason for this switch in grower diets during the postweaning period (d 57–112) was to provide adequate nutrients (i.e., NDF) as calves developed. Grower intake was also measured daily on a pen basis. The nutrient composition of the calf starters, grower #1, and grower #2 for CON and SCFP calves is shown in Table 1.

Every other week, a sample of each feedstuff being fed was collected and frozen at −20°C until analysis. Feed was composited and analysis was performed by Cumberland Valley Analytical Services (Waynesboro, PA). Feeds were analyzed according to AOAC International (2000) for DM (method 930.15), ash (method 942.05), CP (method 990.03), fat (method 954.02 for MR and method 2003.05 for calf starters and growers), ADF (method 973.18), and NDF (Van Soest et al., 1991).

Individual calf BW (Tru-Test; accuracy ± 1%), BCS, hip height (**HH**), and hip width (**HW**) were measured every other week on d 0 (arrival), 14, 28, 42, 56, 70, 84, 98, and 112, and ADG of calves was calculated every other week. Feed efficiency (gain:feed) was calculated preweaning by taking the BW change from d 0 to 56 and dividing it by the total intake from d 0 to 56 of each calf. Postweaning FE was calculated by taking the BW change from d 56 to 112 divided by the total intake from d 56 to 112 of each pen.

Daily fecal and respiratory scores were recorded for each calf through d 56. Fecal scores were measured on a scale of 1 to 5, with 1 being firm/solid and 5 being white/clear liquid, modified from Kertz and Chester-Jones (2004). Overall respiratory status was evaluated on a scale of 0 to 3, with 0 being no sign of respiratory illness and 3 being multiple signs of respiratory illness, including coughing, labored breathing, fever, drooping ears, ocular discharge, or nasal discharge, modified from the Wisconsin calf respiratory scoring chart (McGuirk and Peek, 2014). Medical interventions were recorded for each calf throughout the entire study (d 0–112) and grouped based on illness (digestive, respiratory, and other). Calves were treated for respiratory illness after they showed 2 or more physical symptoms, including ocular or nasal discharge, rapid or raspy breathing, droopy ears, coughing, fever, or refused MR. Calves were treated with florfenicol (Nuflor; Merck Animal Health) or tulathromycin(Draxxin; Zoetis US). Calves were treated for diarrhea with sulfamethoxazole after being given a fecal score of ≥4. Calves with diarrhea and dehydration also received electrolytes as needed. Calves receiving electrolytes were offered 1.89 L at 1200 h; if they refused electrolytes, an esophageal tube feeder was used to administer the remainder of the electrolytes. Other medications included broad-spectrum antibiotics such as enrofloxacin (Baytril Bayer) and ampicillin (Polyflex; Boehringer Ingelheim) and the anti-inflammatory agent dexamethasone (Vet One). These were administered after veterinary recommendation when the calf had an illness not classified as digestive or respiratory related; that is, a joint or navel infection.

A power analysis was performed to calculate the sample size for the primary outcome variable (ADG). Based on data from Harris et al. (2017), with 95% confidence and 80% power, 19 animals per treatment group were needed to detect differences. To account for potential calf mortality during the trial, 30 animals per treatment group were enrolled. Data were analyzed as a completely randomized block design using the Mixed and GLM procedures of SAS (version 9.4; SAS Institute Inc.) with repeated measures, when applicable, using a first-order autoregressive structure that was selected due to the lowest Akaike and Bayesian information criteria. Growth and performance measurements, including BW, BCS, HH, HW, ADG, intake, and FE were analyzed for normality using the Shapiro-Wilk test, and data were normally distributed (W > 0.85). Continuous response variables included STP, BW, BCS, HH, HW, ADG, FE, and intake, and categorical response variables included medical interventions, fecal scores, and respiratory scores. Calf STP, preweaning BW, BCS, HH, HW, ADG, FE, intake, medical interventions, and fecal and respiratory scores were analyzed with calf as the experimental unit (n = 60). The fixed effects included treatment (*T_i_*; CON and SCFP), batch (*Ba_j_*; 1 or 2), block (*Bl_k_*; 1, 2, or 3 within each batch based on initial BW), the interaction between treatment and batch (*TBa*_(_*_ij_*_)_), and the interaction between treatment and block (*TBl*_(_*_ik_*_)_). The random effect of calf nested within treatment (*C_l_*_(_*_i_*_)_) was also included in the model. The model was represented as follows:

*Y_ijkl_* = *µ* + *T_i_* + *Ba_j_* + *Bl_k_* + *TBa*_(_*_ij_*_)_ + *TBl*_(_*_ik_*_)_ + *C_l_*_(_*_i_*_)_ + *e_ijkl_*,

where *Y_ijkl_* is the response variable, *µ* is the overall mean, and *e_ijkl_* is the error. Postweaning BW, BCS, HH, HW, ADG, FE, intake, and medical interventions were analyzed with pen as the experimental unit (n = 12). Postweaning BW, BCS, HH, and HW were analyzed using d 56 measurements as a covariate (Cov). The fixed effects included treatment (*T_i_*; CON and SCFP), batch (*Ba_j_*; 1 or 2), block (*Bl_k_*; 1, 2, or 3 within each batch based on initial BW), the interaction between treatment and batch (*TBa*_(_*_ij_*_)_), and the interaction between treatment and block (*TBl*_(_*_ik_*_)_). The model was represented as follows:

*Y_ijkl_* = *µ* + *T_i_* + *Ba_j_* + *Bl_k_* + *TBa*_(_*_ij_*_)_ + *TBl*_(_*_ik_*_)_ + Cov + *e_ijkl_*,

where *Y_ijkl_* is the response variable, *µ* is the overall mean, and *e_ijkl_* is the error. A *P*-value ≤0.05 was determined to be statistically significant and a *P*-value >0.05 and <0.10 was determined to indicate a statistical tendency.

Calf STP, initial (d 0), weaning (d 56), and final (d 112) body measurements and pre- and postweaning ADG and FE of the calves based on study treatment are presented in Table 2. No treatment effect was observed for STP, BW, BCS, HH, or HW at d 0 or 56 (*P* ≥ 0.31).

**Table 2:** Serum total protein (STP), initial (0 d) and weaning (56 d) body measurements, preweaning (0–56 d) ADG and feed efficiency (FE) of Holstein bull calves fed either the control (CON; n = 30 calves) or *Saccharomyces cerevisiae* fermentation products (SCFP; n = 30 calves) treatment diet, and final (112 d) body measurements and postweaning (57–112 d) ADG and FE based on treatment diets (CON; n = 6 pens vs. SCFP; n = 6 pens)

Items	Treatment^1^		SEM	*P*-value		
	CON	SCFP		Treatment	Batch	Block
STP, mg/dL	5.95	6.04	0.11	0.55	0.007	0.19
Preweaning						
BW, kg						
0 d	45.2	45.0	0.4	0.81	0.72	<0.001
56 d	85.4	84.5	1.5	0.67	0.39	0.04
BCS^2^						
0 d	2.22	2.22	0.01	0.80	0.99	0.001
56 d	2.88	2.84	0.03	0.31^3^	0.04	0.12
Hip height, cm						
0 d	82.7	83.1	0.4	0.49	0.56	<0.001
56 d	95.3	95.7	0.5	0.52	0.60	<0.001
Hip width, cm						
0 d	16.8	16.6	0.1	0.34	0.96	<0.001
56 d	21.4	21.3	0.2	0.62^4^	0.67	0.02
ADG, kg/d	0.71	0.71	0.03	0.95	0.45	0.78
FE,^5^ kg/kg	0.52	0.52	0.01	0.87	<0.0001	0.04
Postweaning						
BW, kg						
112 d	147.2	153.6	1.4	0.05	0.25	0.06
BCS^2^						
112 d	3.46	3.46	0.006	0.76	0.0005	0.009
Hip height, cm						
112 d	107.4	107.2	0.2	0.69^6^	0.00	0.02
Hip width, cm						
112 d	26.8	27.4	0.1	0.01	0.10	0.06
ADG, kg/d	1.12	1.22	0.03	0.07	0.56	0.06
FE,^5^ kg/kg	0.29	0.30	0.002	0.02^7^	0.005	0.003

1 CON = 24% CP:17% fat MR, calf starter, and calf grower with no SCFP added; SCFP = 24% CP:17% fat MR with SmartCare (Diamond V), calf starter with NutriTek (Diamond V), and calf grower with NutriTek.

2 BCS was measured on a scale of 1 to 5.

3 Treatment × block interaction tendency (*P* = 0.08).

4 Treatment × batch interaction (*P* = 0.04).

5 FE = BW gain:feed intake.

6 Treatment × batch interaction tendency (*P* = 0.08).

7 Treatment × block interaction (*P* = 0.04).

There was no treatment effect for preweaning ADG or FE (*P* ≥ 0.87). This is similar to results from Alugongo et al. (2017), who did not observe treatment effects on preweaning ADG or FE, and from Lesmeister et al. (2004), who reported no treatment effects of SCFP on preweaning FE. Other studies, however, have reported that SCFP increased the growth rate of calves challenged with *Salmonella* 2 wk after being supplemented with SCFP (Brewer et al., 2014) and increased final BW (d 42) of calves that were not challenged (Lesmeister et al., 2004).

No treatment effect was observed for BCS or HH at d 112 (*P* ≥ 0.69). However, BW and HW were increased in SCFP calves at d 112 (*P* ≤ 0.05). A treatment tendency was observed for postweaning ADG (*P* = 0.07), with SCFP calves having greater ADG than CON calves (1.22 vs 1.12 kg/d, respectively). This agrees with previous research that found a significantly greater postweaning ADG when SCFP is supplemented (Lesmeister et al., 2004). This may suggest that SCFP helps to improve the growth of calves after experiencing a stress event (e.g., morbidity, heat stress, and weaning), which agrees with prior research (Lesmeister et al., 2004; Brewer et al., 2014). Supplementation with SCFP has been shown to increase rumen development, through longer papillae length and greater papillae width (Lesmeister et al., 2004; Brewer et al., 2014). Therefore, this increase in ADG may be explained by improved rumen development and absorption capability in the SCFP calves (Xiao et al., 2016). Calves fed SCFP also had improved postweaning FE compared with CON (*P* = 0.02), which is consistent with the increased ADG postweaning in SCFP calves that was observed. No prior studies have evaluated FE in a group setting after weaning; therefore, the improved postweaning FE observed in SCFP calves in the current study warrants further validation. However, improved FE after weaning could imply that SCFP is able to mitigate the negative effects caused by stress through improved rumen development and absorption efficiency (Xiao et al., 2016). Also, various batch and block effects were observed for STP, BW, BCS, HH, HW, ADG, and FE (*P* < 0.04; Table 2), indicating the effects of initial size of calves and environment also affected the performance of calves.

Milk replacer intake, starter intake, individual intake, group intake, medical interventions, fecal scores, and respiratory scores based on study treatment are presented in Table 3. No treatment differences were observed for MR intake, starter intake, individual intake, or group intake (*P* ≥ 0.30). In agreement with the current study, Alugongo et al. (2017) did not observe differences in starter intake when SCFP was supplemented to calves; however, they did report low overall intake due to an increased MR feeding rate. Lesmeister et al. (2004) did observe that SCFP calves had greater starter intakes, but it is worth noting that those calves were weaned at approximately 35 d of age. A batch effect was observed for starter intake and individual intake (*P* = 0.0002), with that of batch 2 being greater than that of batch 1. A tendency for a treatment × batch interaction was observed for group intake (*P* = 0.07), which was greater for CON calves in batch 1 and greater for SCFP calves in batch 2.

**Table 3:** Preweaning intake, medical interventions, fecal scores, and respiratory scores of Holstein bull calves fed either the control (CON; n = 30 calves) or *Saccharomyces cerevisiae* fermentation products (SCFP; n = 30 calves) treatment diet from d 0 to 56, as well as postweaning intake and medical interventions based on treatment diets (CON; n = 6 pens vs. SCFP; n = 6 pens) from d 57 to 112

Item	Treatment^1^		SEM	*P*-value		
	CON	SCFP		Treatment	Batch	Block
Preweaning						
Milk replacer intake, kg	42.05	42.05	0.02	0.95	0.14	0.30
Starter intake, kg	35.06	35.15	2.17	0.98	0.0002	0.52
Individual intake,^2^ kg	77.11	77.20	2.17	0.98	0.0002	0.52
Medical intervention^3^						
Digestive	0.52	0.46	0.11	0.71	0.86	0.24
Respiratory	0.41	0.31	0.11	0.51	0.62	0.69
Other	0.17	0.04	0.07	0.20	0.57	0.73
Fecal score^4^	2.12	2.15	0.02	0.38	0.36	0.70
Respiratory score^5^	0.14	0.12	0.02	0.38	<0.0001	0.70
Postweaning						
Group intake,^6^ kg/calf	220.8	228.3	5.59	0.30^7^	0.20	0.74
Medical intervention						
Respiratory	0.58	0.00	0.10	0.02	0.12	0.07

1 CON = 24% CP:17% fat MR, calf starter, and calf grower with no SCFP added; SCFP = 24% CP:17% fat MR with SmartCare (Diamond V), calf starter with NutriTek (Diamond V), and calf grower with NutriTek.

2 Total individual intake = milk replacer intake + starter intake.

3 Medical interventions were calculated based on the total number of medical interventions in each category (digestive, respiratory, or other) based on study treatment/number of calves on treatment diets.

4 Fecal scores were assigned on a scale of 1 to 5, only recorded until d 56 (average daily score).

5 Respiratory scores were assigned on a scale of 0 to 3, only recorded until d 56 (average daily score).

6 Group intake = individual pen intake/number of calves in pen.

7 Treatment × batch interaction tendency (*P* = 0.07).

Poor calf health can compromise growth and future productivity and increase treatment costs. In this study, no treatment effects were observed for the number of medical interventions based on digestive, respiratory, or other illnesses (i.e., not digestive or respiratory related) before weaning, fecal score, or respiratory score (*P* ≥ 0.20). It has previously been reported that SCFP reduced diarrhea and improved fecal scores in calves (Lesmeister et al., 2004; Magalhães et al., 2008). However, a treatment effect was observed for respiratory treatments that occurred postweaning (*P* = 0.02), with SCFP calves being treated less frequently than CON calves. Few studies feeding SCFP to calves have evaluated the frequency of respiratory illness. Recently, Mahmoud et al. (2020) evaluated immune parameters, respiratory disease–related clinical signs, and gross lung pathology in control and SCFP-supplemented calves that had been challenged with bovine respiratory syncytial virus. The SCFP calves had fewer cases of secondary infection, respiratory clinical disease, and lung pathology following the viral challenge. It is possible that SCFP supplementation enhances the innate immune function of calves while also regulating the immune reaction in the lungs to reduce damage or consolidation and expedite recovery. Again, illness rate inconsistencies between different studies could be due to environmental and seasonal differences, the type of SCFP used, and the level of pathogenicity. Additionally, a batch effect was seen for respiratory score (*P* < 0.0001), which was increased in batch 1 compared with batch 2. A tendency for a block effect was observed for postweaning respiratory treatments (*P* = 0.07), with block 1, or the lightest BW calves, having a higher incidence of respiratory treatments.

In conclusion, supplementation with SCFP improved ADG, FE, BW, and HW in dairy calves postweaning. The negative growth effects associated with stress events such as weaning may have been minimized in calves receiving SCFP. Feeding calves SCFP also reduced the incidence of respiratory disease intervention postweaning, thereby reducing antibiotic use and potentially improving the health status of calves after weaning.
